# Multiple introductions and secondary dispersion of *Tubastraea* spp. in the Southwestern Atlantic

**DOI:** 10.1038/s41598-019-50442-3

**Published:** 2019-09-27

**Authors:** K. C. C. Capel, J. Creed, M. V. Kitahara, C. A. Chen, C. Zilberberg

**Affiliations:** 1 Departamento de Zoologia, Universidade Federal do Rio de Janeiro, Rio de Janeiro, Brazil; 2 Associate Researcher, Coral-Sol Research, Technological Development and Innovation Network, Rio de Janeiro, Brazil; 3 Centro de Biologia Marinha, Universidade de São Paulo, São Sebastião, Brazil; 4 Departamento de Ecologia, Universidade do Estado do Rio de Janeiro, Rio de Janeiro, Brazil; 5 Departamento de Ciências do Mar, Universidade Federal de São Paulo, Santos, Brazil; 6 Biodiversity Research Center, Academia Sinica, Taipei, Taiwan; 7 Instituto de Biodiversidade e Sustentabilidade, Rio de Janeiro, Brazil

**Keywords:** Invasive species, Molecular ecology, Population genetics

## Abstract

Accidental introduction through ballast water and biofouling are currently the main factors responsible for spreading non-indigenous species in the marine realm. In the Southwestern Atlantic, two scleractinian corals, *Tubastraea coccinea* and *T. tagusensis*, have been introduced by opportunistic colonization in 1980 and are now widespread along more than 3,500 km of coastline. To better understand the invasion process and the role of vectors in spreading these species, we sampled 306 and 173 colonies of *T. coccinea* and *T. tagusensis* from invaded sites, possible vectors and one native population. Analyses revealed a higher diversity of multi-locus genotypes (MLGs) on vectors, suggesting that they were contaminated prior to their arrival in the Southwestern Atlantic, and a high proportion of clones at invaded sites, with few genotypes spread over ~2,000 km. This broad distribution is most likely a result of secondary introductions through the transport of contaminated vectors. Results also suggest the occurrence of multiple invasions, mainly in the northernmost sites. In summary, clonality, secondary introductions, and multiple invasions are the main reasons for the broad spread and invasive success of *Tubastraea* spp. in the Southwestern Atlantic. Consequently, the correct control of vectors is the most effective approach for management and prevention of new invasions.

## Introduction

Marine bioinvasion is reshaping the distribution and biogeographic patterns of species worldwide and is reaching unprecedented levels with hundreds of species being transported to new environments every year^1–4^. Accidental introductions can occur through a number of ways, such as aquaculture, trade in ornamental species, canals linking previously unconnected waters, ballast water and biofouling, the last two being the main factors responsible for spreading non-indigenous species in the marine realm^2,5–7^. As a consequence of the increasing marine traffic, vessels (e.g. cargo ships, oil platforms, floating docks, buoys; herein called vectors) transport a large number of species (either by ballast water or biofouling), some of which will be able to establish and disperse, becoming invasive in the new environment^6,8^.

Recently, a review listed 15 non-indigenous species causing major negative impacts in the South Atlantic, including two azooxanthellate corals (*Tubastraea coccinea* and *T. tagusensis*)^9^. *T. coccinea* was first reported in the Atlantic during the 1940’s in Curaçao and Puerto Rico, and was probably introduced as biofouling on ship hulls traveling from Indo-Pacific waters^10–12^. Further records of the genus were reported in the Northwestern Atlantic in 2004 (*T. coccinea*)^13^, Southwestern Atlantic in the 1980’s (*T. coccinea* and *T. tagusensis*)^14,15^ and Gulf of Mexico in 2010 (*T. coccinea* and *T. micranthus*)^16^. Given the pattern of sea surface currents and previous examples from other invasive species, the Gulf of Mexico and Florida could have been naturally invaded by Caribbean populations^12^. However, the occurrence of *T. micranthus*, a species not yet found in the Caribbean, on oil platforms at Gulf of Mexico indicates that human vectors have also been responsible for the introduction into this region^17^. In the Southwestern Atlantic, *Tubastraea* spp. were firstly reported as fouling on offshore oil platforms in Rio de Janeiro State^12,14^. The genus is now widespread on rocky shores and artificial substrates (oil platforms, buoys, wrecks, piers, and drillships) along more than 3,500 km, from Ceará (02°29′S, 39°51′W)^18^ to Santa Catarina (27°17′S, 48°22′W)^19^, outcompeting native and endemic species^20–24^. Although there is no doubt that the introduction in the Southwestern Atlantic was through biofouling^12^: (1) the invasion history remains unclear; (2) there are no studies elucidating why these two species have been so successful in invading the Brazilian coast; and (3) the role of vectors in spreading them along the coast is unknown.

Successful invasive species frequently share a set of life history and ecological traits that facilitate their establishment, such as rapid growth rate, large number of offspring (r-selected species), sexual and asexual reproduction, early maturity and phenotypic plasticity^25^. When combined with high propagule pressure, a measure of the number of individuals released and the number of release events, the chances of survival in a new environment are considerably enhanced^26,27^. *Tubastraea* spp. possesses all such traits^28–33^, which may have facilitated their successful establishment and dispersal in the Southwestern Atlantic. Furthermore, considering their reproductive biology and rapid expansion along the Brazilian coastline, it is highly likely that vectors have been playing a key role in *Tubastraea* spp. dispersion along the Southwestern Atlantic^34^. Indeed, *Tubastraea* spp. have been recorded on at least 23 vectors, some of which have been towed along the Brazilian coast without biofouling control^12^.

The occurrence of multiple introductions has also been correlated with invasion success^35–40^. When founded by a small number of individuals, recently established populations can suffer a drastic reduction in genetic diversity as a consequence of genetic drift. Ultimately, genetic drift can have negative consequences such as the fixation of deleterious alleles and decrease of the species resilience^25,41–45^. On the other hand, multiple introduction events of non-indigenous species from more than one native population can lead to an increase in genetic diversity by mixing previously separated populations and increasing the propagule pressure, consequently reducing negative genetic outcomes of the invasion process and enhancing the possibility of a successful invasion^7,26,27,36,40,46,47^.

A better understanding of the invasion process and the ways by which invasive species are spreading into new environments is essential for improving management effectiveness and control plans that ultimately reduce or prevent future invasions^6,25,48^. Here we use a set of microsatellite markers^34^ to: (1) investigate genetic diversity and clonality at invaded sites and on vectors along the Brazilian coast; (2) provide insights into the role of vectors at spreading these invasive corals in the Southwestern Atlantic; and (3) evaluate the population structure at invaded sites with regard to the possibility of multiple introduction events.

## Results

### Clonality

**Figure 1:**
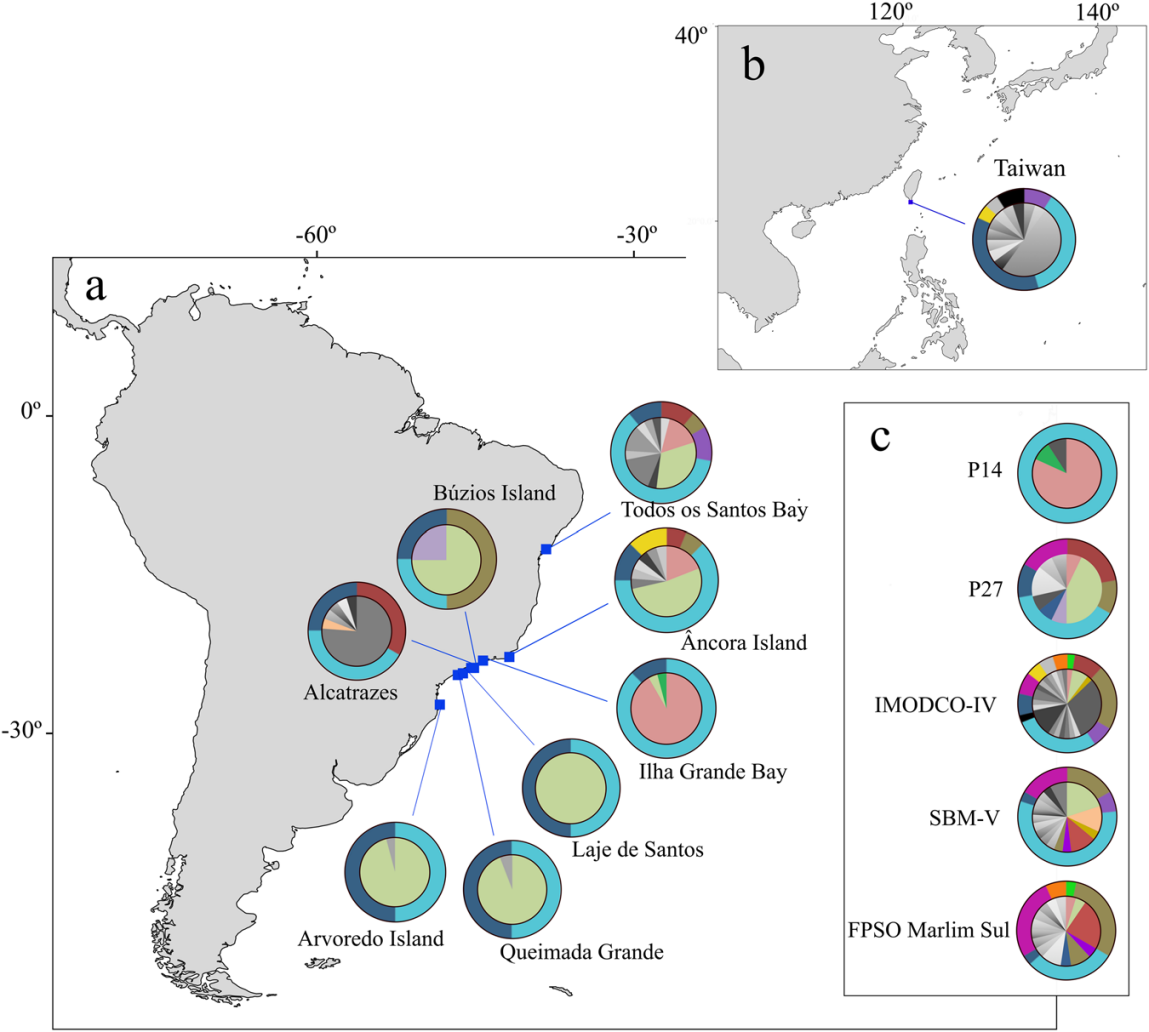
Sampling sites for *T. coccinea* (**a**) along the invasive range in Southwestern Atlantic, (**b**) at a native population in Taiwan and (**c**) at five vectors located on the Brazilian coast. Pie diagrams show the number of multilocus genotypes (MLGs) (inner circle) and the allele frequency of the locus Tco 29 (with a total of 13 alleles, outer circle) per population. Colors indicate MLGs or alleles that are shared among two or more sites and gray scale indicate MLGs and alleles that are exclusive for the correspondent site (not observed on any other analyzed site). The locus Tco 29 is the second most diverse for *T. coccinea* and was chosen to exemplify allele sharing among invaded sites, vectors and Taiwan.

**Figure 2:**
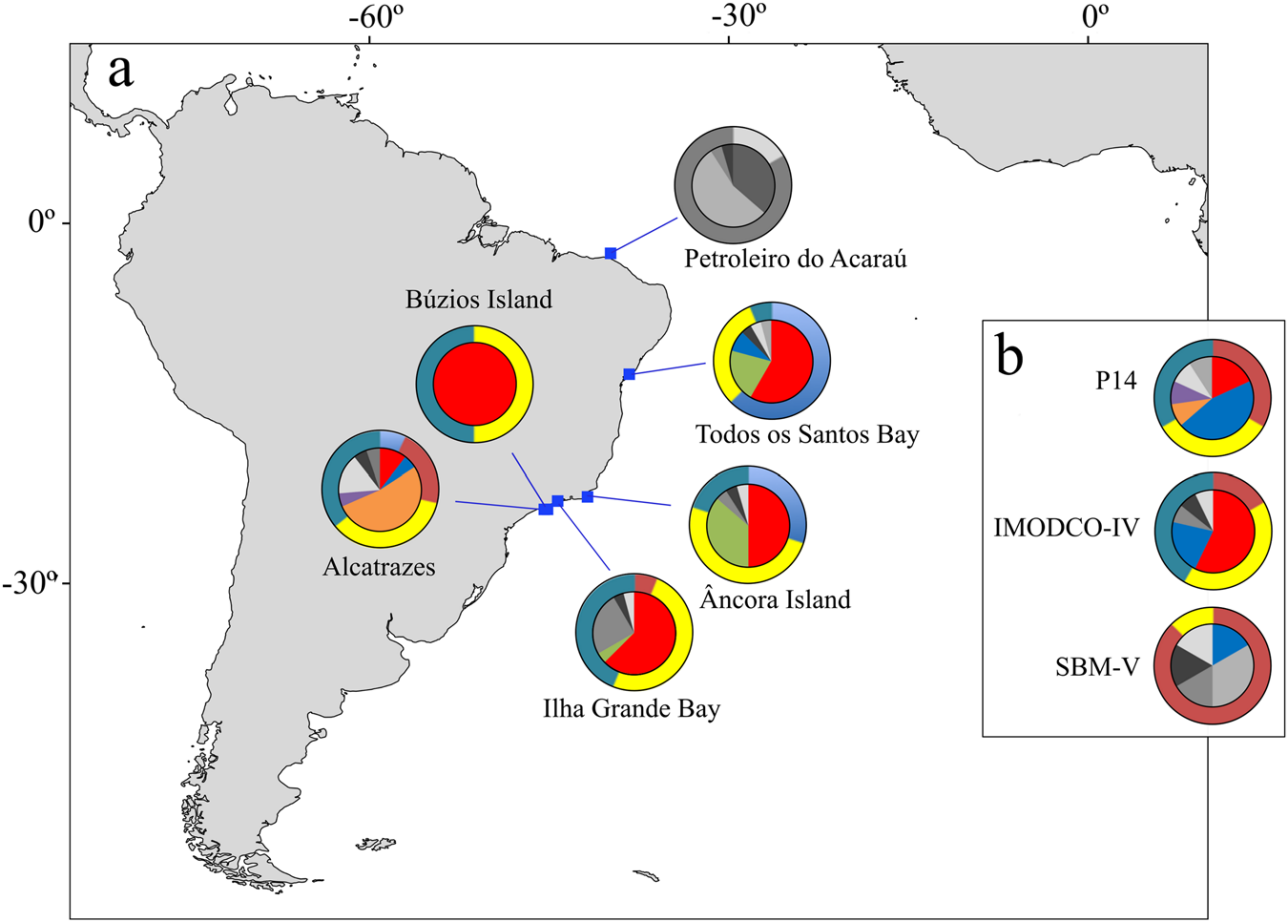
Sampling sites for *T. tagusensis* (**a**) along the invasive range in Southwestern Atlantic and (**b**) at three vectors located on the Brazilian coast. Pie diagrams show the number of multilocus genotypes (MLGs) (inner circle) and the allele frequency of the locus Tco 34 (with a total of six alleles, outer circle) per population. Colors indicate MLGs or alleles that are shared among two or more sites and gray scale indicate MLGs and alleles that are exclusive for the correspondent site (not observed on any other analyzed site). The locus Tco 34 is the second most diverse for *T. tagusensis* and was chosen to exemplify allele sharing among invaded sites and vectors.

**Figure 3:**
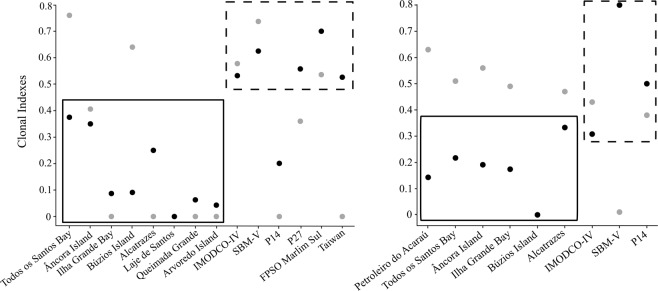
Clonal indicess for (**a**) *T. coccinea* and (**b**) *T. tagusensis*. Black dots indicate the clonal richness (R), ranging from 0 to 1, when all samples analyzed correspond to a different MLG and gray dots indicate the genotypic evenness (V), ranging from 0 to 1, when genets each have the same number of ramets. Continuous and dotted rectangles indicate invaded sites and vectors (plus native Taiwanese population), respectively.

Analyses revealed a high proportion of clones for both *T. coccinea* and *T. tagusensis* with only 84 (28%; N = 298) and 30 (18%; N = 166) unique MLGs within all sampled sites. Similar proportion of clones were found when all indivuduals with missing data were removed (*T. coccinea*: N = 250, MLG = 60 or 20%; *T. tagusensis*: N = 156, MLG = 22 or 13%). For *T. coccinea*, invaded sites had, in general, less MLGs when compared to vectors and native population, with five sites holding three or less MLGs (Table 1, Fig. 1). The same was not observed for *T. tagusensis*, for which the highest number of MLGs observed was at an invaded site (Alcatrazes: MLG = 7, Table 1, Fig. 2). The existence of two MLGs for *T. coccinea* at the invaded sites Queimada Grande and Santa Catarina is probably a consequence of missing data in one locus and these two populations are likely dominated by only one MLG. For *T. coccinea*, clonality was higher at invaded sites contrasting to vectors and native population (Fig. 3a). The genotypic evenness did not show a clear pattern, with five invaded sites, one vector and the native site being dominated by one genotype. However, the remaining sites (two invaded sites and three vectors) had more equitable distribution of ramets among the observed MLGs (genotypic evenness (*V*) close to 1, Fig. 3a). Interestingly, the same pattern for MLGs and clonal richness was not as clear for *T. tagusensis*. Invaded sites and vectors had similar numbers of MLGs (except for Búzios Island, with one MLG) and clonal richness was slightly lower on vectors, reaching 0.8 in the vector SBM-V (Fig. 3b). The genotypic evenness did not change between invaded sites and vectors for this species (Fig. 3b).

**Table 1:** Summary of statistics per samples site for the species *Tubastraea coccinea* (N = 298) and *T. tagusensis* (N = 166).

Status	Site	N	MLG	Psex	A	Ar	Ae	Ho	He	*F*_IS_
***Tubastraea coccinea***										
Invaded	Todos os Santos Bay (TSB)	25	10	0	22	2.2	3	0.58	0.55	−0.01
	Âncora Island (AI)	21	8	0	20	2.2	2	0.50	0.54	0.13
	Ilha Grande Bay (IGB)	24	3	1	13	1.8	0	0.50	0.39	−0.16
	Búzios Island (BI)	12	2	1	14	2.2	0	0.54	0.42	−0.15
	Alcatrazes (Alc)	21	6	0	20	2.4	1	0.57	0.52	0.02
	Laje de Santos (LS)	24	1	2	11	1.8	0	0.83	0.42	−1
	Queimada Grande (QG)	17	2	1	11	1.8	0	0.83	0.42	−1
	Arvoredo Island (ArI)	24	2	2	11	1.8	0	0.83	0.42	−1
Vectors	IMODCO-IV	39	21	0	41	2.8	8	0.54	0.66	0.21*
	SBM-V	25	16	0	23	2.2	0	0.41	0.48	0.18
	P14	11	3	1	11	1.7	0	0.42	0.32	−0.18
	P27	14	8	0	23	2.5	0	0.54	0.57	0.12
	FPSO Marlim Sul	21	15	0	27	2.3	3	0.42	0.61	0.34*
Native	Taiwan	20	11	0	22	2.2	4	0.39	0.49	0.25
***Tubastraea tagusensis***										
Invaded	Petroleiro do Acaraú (PA)	22	4	2	14	1.8	11	0.67	0.35	−0.91
	Todos os Santos Bay (TSB)	24	6	2	17	1.9	0	0.53	0.35	−0.49
	Âncora Island (AI)	22	5	0	19	2.0	4	0.50	0.42	−0.07
	Ilha Grande Bay (IGB)	24	5	3	14	1.7	0	0.58	0.32	−0.81
	Búzios Island (BI)	24	1	2	13	1.6	0	0.62	0.31	−1
	Alcatrazes (Alc)	19	7	2	16	1.9	2	0.62	0.36	−0.69
Vectors	IMODCO-IV	14	5	1	18	1.9	2	0.65	0.41	−0.51
	SBM-V	6	5	0	20	2.2	5	0.46	0.45	0.09
	P14	11	6	0	20	2.1	4	0.57	0.41	−0.30

N = number of sampled individuals, MLG = number of unique multilocus genotypes per site, Psex = number of individuals with Psex ≥ 0.01, A = number of alleles, Ar = allelic richness, Ae = number of exclusive alleles, Ho = observed heterozygosity, He = expected heterozygosity. *Indicates significant deviations from Hardy-Weinberg equilibrium.

Of the observed MLGs, ten (12%) from *T. coccinea* and five (17%) from *T. tagusensis* are shared with one or more sampled sites (Figs 1 and 2). *T. coccinea* had five of its MLGs shared among invaded sites and vectors, while the remaining five were shared exclusively among vectors. Vectors also showed a higher number of exclusive MLGs, not shared with any other sampled site (Table 1; Fig. 1). Neither the invaded sites nor the vectors shared any MLGs with the native population from Taiwan. One MLG was found on three vectors and all invaded sites (except for Alcatrazes), indicating the occurrence of clones separated by over 1,900 km along the Brazilian coast (Fig. 1). Although for *T. coccinea* no MLG was shared between Alcatrazes and other invaded sites, the predominant MLG found at this site differed by only one allele from the predominant MLG found at other invaded sites (green MLG showed at the inner circle in Fig. 1). Similarly, for *T. tagusensis*, the predominant MLG found at Alcatrazes also differed by only one allele from the predominant MLG found at other invaded sites (red MLG showed at the inner circle in Fig. 2). Of the five MLGs observed for *T. tagusensis*, four were shared between at least one vector and one invaded site and one was found exclusively at invaded sites. The northernmost sampled site, Petroleiro do Acaraú, did not share MLGs with any other invaded site or vectors.

### Genetic diversity

For the genetic diversity analyses, all clones found within populations were removed and only one representative of each MLG was included, additionally to those individuals with *P*_sex_ value higher than 0.01 (Table 1). Within the analysed data set, a total of eight individuals of *T. coccinea* (3%) and 12 (7%) *T. tagusensis* had *P*_sex_ value higher than 0.001, thus being considered products of distinct sexual reproduction events. Two loci from *T. coccinea* (Tco 1 and Tco 30) and two from *T. tagusensis* (Tco 4 and Tco 37) showed evidence of linkage disequilibrium with at least two other loci and were excluded from further genetic diversity analyses. There was no evidence of null alleles for *T. coccinea*, while for *T. tagusensis* three loci showed evidence of null alleles for one population each (Supplementary Table S1).

For *T. coccinea*, the number of alleles and allelic richness ranged from 11 to 41 and 1.7 to 2.8, respectively. For this species, exclusive alleles were found at three invaded sites (Todos os Santos Bay, Âncora Island and Alcatrazes), two vectors (SBM-V Araça and FPSO Marlim Sul) and in the native population (Taiwan) (Table 1). The frequency distribution of alleles for one locus (Tco 29, with 13 alleles – Fig. 1, outer circle) shows that invaded sites, vector and the native population share four alleles and that the number of exclusive alleles is higher on vectors and in the native population. All but Tco 5 has at least one allele shared among all sites, and Taiwan shares at least one allele per locus with one or more invaded or vector sites (Supplementary Table S2). Observed (Ho) and expected (He) heterozygosity ranged from 0.39 to 0.83 and 0.32 to 0.66, respectively (Table 1). Only the vectors IMODCO-IV and FPSO Marlim Sul had significant deficits of heterozygosity (Table 1). Although not significant, many sites (e.g., LS, QG and ArI; Table 1) had Ho values twice as high as He, indicating heterozygote excesses. However, it is important to note that these values need to be interpreted with caution, since all sites that displayed Ho excess had low number of individuals analysed. The inbreeding coefficient (*F*is) was negative for most invaded sites (except for AI and Alc) and P14, indicating an excess of heterozygotes (Table 1). The remaining vectors and the native population had inbreeding coefficients ranging from 0.12 (i.e. P27) to 0.34 (i.e. FPSO Marlim Sul).

For *T. tagusensis*, the number of alleles and allelic richness were similar among sampled sites, ranging from 13 to 20 and 1.6 to 2.2, respectively. Exclusive alleles were found at all but three invaded sites (TSB, IGB and BI), with the highest number observed at the northernmost site, Petroleiro do Acaraú (Ae = 11; Table 1). Figure 2 shows the frequency distribution of alleles for the locus Tco 34 (outer circle), with all sites but Petroleiro do Acaraú sharing at least one allele. Five out of eight loci had shared alleles among Petroleiro do Acaraú and at least one other site and only three loci had shared alleles among all samples sites (Supplementary Table S2). Observed heterozygosity (Ho) (ranging from 0.46 to 0.67) was higher than the expected (He) (ranging from 0.31 to 0.45) in all sites (Table 1), with no significant deficits of heterozygosity. The inbreeding coefficient (*F*is) was negative for all sites but SBM-V, indicating an excess of heterozygotes (Table 1).

### Population structure

**Figure 4:**
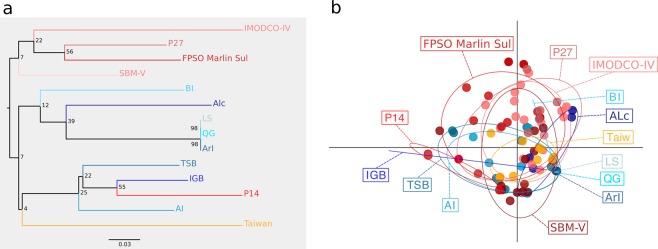
(**a**) Neighbor-joining (NJ) tree based on Cavalli-Sforza’s and Edwards chord distance and (**b**) Principal Components Analysis (PCA) for *T. coccinea*.

For *T. coccinea*, the NJ had low support values and PCA analysis showed that 95% confidence ellipses of each site were clearly overlapping, revealing no difference in overall gentic variance amond sites (Fig. 4b). Bayesian clustering analysis also did not recover any clear genetic cluster for any invaded site, vector or the native population of *T. coccinea* (Supplementary Fig. S1). The two methodologies used to estimate *K* gave similar results of five, eight or nine possible genetic clusters (Supplementary Fig. S1).

**Figure 5:**
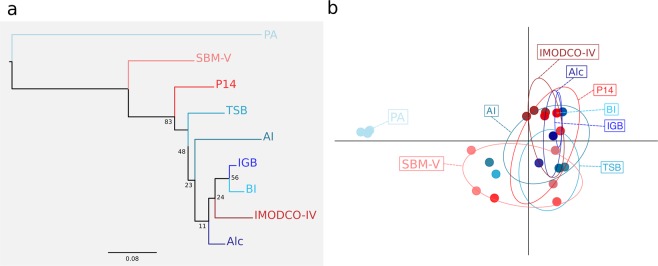
(**a**) Neighbor-joining (NJ) tree based on Cavalli-Sforza’s and Edwards chord distance and (**b**) Principal Components Analysis (PCA) for *T. tagusensis*.

For *T*. *tagusensis*, NJ analysis and PCA recovered Petroleiro do Acaraú as the most divergent site (first axis of PCA explaining 53% of the variance), while all remaining sites overlapped, indicating no clear difference in overall gentic variance amond them (Fig. 5b). Bayesian analysis recovered three genetic clusters (Supplementary Fig. S3), suggesting that the Southwestern Atlantic was colonized by more than one native population, as observed for *T. coccinea*. Except for Petroleiro do Acaraú, the observed genetic clusters were not a function of population structure between localities (Supplementary Fig. S3). No substructure was observed when analyzing higher *K*. Evidence of interbreeding between two clusters could be observed at Todos os Santos Bay, Alcatrazes and IMODCO-IV (Supplementary Fig. S3).

## Discussion

Forty years after its first record, the genus *Tubastraea* has spread its range over 3,500 km along the Brazilian coast with increasing densities^21,49^. Here we show evidence that *T. coccinea* and *T. tagusensis* were introduced in the Southwestern Atlantic Ocean in more than one event. Furthermore, our results point to a critical role of the past transport of oil platforms from the Indo-Pacific in introducing these species in the Southwestern Atlantic Ocean, and that buoys and Floating Production Storage and Offloading Vessels (FSPOs) that travel from offshore oil platforms to onshore localities are acting as vectors in spreading these species along the Brazilian coast.

For both *T. coccinea* and *T. tagusensis* the Southwestern Atlantic invaded sites have a higher proportion of clones when compared to vectors, with sites dominated by a single genotype (Figs 1 and 2, see also^34^). Thus, their early reproductive age^33^ and ability to reproduce asexually are probably the main factors responsible for their success as invaders. When environmental conditions are suitable, such abilities enable large densities from a small starting number of individuals. Interestingly, although *T. coccinea* from Taiwan had fewer clones when compared to the majority of the Brazilian invaded sites, half of the examined samples were composed of clones of the same genet. This demonstrates that asexual reproduction also plays an important role in its native distribution range. As discussed by previous studies, asexual reproduction in *Tubastraea* occurs through the asexual production of larvae^29,34^, a strategy broadly used by anthozoans^50–52^, but to date only observed in three scleractinian species, *T. coccinea*, *T. diaphana* and *Pocillopora damicornis*^29,53,54^.

Along the Southwestern Atlantic, *T. coccinea* and *T. tagusensis* had one dominant genotype each (shared by 36% and 46% of all analyzed individuals, respectively), showing an over-representation of few genotypes. This may be a consequence of the invasion process where only one or a few genotypes better fitted to the recently invaded environment were able to establish and disperse^55,56^. A similar pattern was previously observed for *Pocillopora damicornis* in Hawaii^55^ and Reunion Islands^57^, and also for *Acropora palmata* in the French Antilles^58^. Nevertheless, those studies analyzed samples collected over small geographic scales (less than 20 km), while the dominant genotype of *T. coccinea* and *T. tagusensis* in invaded sites were spread over 1,500 and 2,000 km, respectively (Figs 1 and 2). Three non-exclusive hypotheses could explain such broad clonal distribution: (1) long-distance dispersal of asexual larvae; (2) multiple events of introduction from the same native population; or (3) secondary introduction from one invaded site to another.

Although possible for nearby localities, larval dispersal itself does not explain the broad distribution of clones at sites separated by more than 1,500 km. *Tubastraea* has a gregarious settlement behavior and most larvae settle within 1 to 3 days^30^, even though experiments in aquaria have shown that larvae can be competent for about 18 days^30,33^ and another observation mention competent *T. coccinea* larvae after 100 days (Richmond, pers. comm.^59^). Nevertheless, due to the Brazilian surface currents regime, it would be oceanographically not probable that competent larvae would travel long distances. Furthermore, the gregarious settlement with high local clonality observed for *Tubastraea* follows the “strawberry-coral” model^60^, when organisms use sexual reproduction to disperse genotypically diverse individuals, while asexual reproduction helps to spread locally adapted genotypes. Interestingly, an opposite hypothesis of dispersal capability was proposed for *P. damicornis*, where the asexual larvae would be responsible for long-distance dispersal^61^. Indeed, studies showing evidence of local recruitment from sexual larvae with low clonality corroborate the hypothesis that asexually produced larvae of *P. damicornis* may travel further^62,63^. Future studies comparing the size and duration of sexual and asexual larvae of *Tubastraea* spp. *in situ* are recommended to test if they follow such pattern.

The second and third hypotheses are the most likely explanations for the observed clonal distribution. The first record of *Tubastraea* spp. in the Southwestern Atlantic was on offshore oil platforms from the Campos Basin, north of Rio de Janeiro State, in the late 1980s. In such context, oil platforms built by foreign companies abroad that were slow towed to the Southwestern Atlantic may have been colonized with local fauna that were further transported as biofouling. Thus, such structures are the most probable means of introduction of these species along the Brazilian waters^12^. Once the platforms started to operate, their associated structures (such as buoys) and the year around movement of associated platforms and offshore support fleet shorewards are the most probable vectors for spreading *Tubastraea* spp. along the Brazilian coast. Indeed, the distributional range of *Tubastraea* spp. in the Southwestern Atlantic appears to be directly associated to sites with intense ship traffic and waterway terminals. de Paula and Creed^64^ analyzed the distribution and expansion of *Tubastraea* spp. at Ilha Grande Bay and found that the Petrobras oil terminal or Verolme shipyard were likely points of introduction of these species at that locality. In addition, Ferreira *at el*.^65^ found 22 non-indigenous species when analyzing drill-ships, platforms and cargo ships in Arraial do Cabo, including *T. coccinea*, and other studies had demonstrated that artificial substrates facilitate invasion^66–68^. *Tubastraea* spp. seem to be opportunistic and have been reported on artificial substrates at both invaded^12–14,65^ and native localities^69^. To confirm the hypothesis of multiple introduction events from the same native population it is essential to know the origin of the vessels and when they first arrived in the Southwestern Atlantic. However, this information is not easily traceable and could not be verified. Of the five vectors included in the present study, such information was traceable for two: P27 was brought from Singapore to Arraial do Cabo, Brazil, in 1998; and P14 was built in France in 1983, with no information of where or when it has first arrived in Brazil. Singapore is within the natural distributional range of *Tubastraea* spp.^70^ and it is highly possible that the platform P27 was infested before its arrival in the Atlantic. Indirect evidence supporting this assumption is the large size of colonies sampled on the platform and the first records of *Tubastraea* spp. in Arraial do Cabo only one-year after the arrival of P27 in the region^12^. Regarding the platform P14, it probably arrived free from *Tubastraea*, as there are no records of this genus in Europe yet. However, it was probably contaminated in Brazilian or Caribbean waters and then became a vector. Although we cannot confirm the origin of all vectors, the (i) occurrence of unique MLGs, (ii) exclusive alleles not found within invaded sites (Figs 1 and 2), and (iii) the higher number of alleles and clonal richness observed at all analyzed vectors (except for P14) compared to invaded sites, suggest that those vectors were already contaminated with *Tubastraea* spp. prior to their arrival in the Southwestern Atlantic.

The occurrence of secondary introduction from one invaded site to another is supported by information of vessels being transported along the Brazilian coast (Table 2)^12^ and the occurrence of clones on both invaded sites and vectors. The platforms P14 and P27 and the monobuoy IMODCO-IV were transported at least once along the Brazilian coast after being contaminated (Table 2)^12^. Furthermore, the traffic of FSPOs from contaminated platforms to onshore terminals might have acted as key vectors as well. Although *Tubastraea* spp. have been previously recorded at all cited sites prior to the arrival of the analyzed vectors, the transport of contaminated vectors further supports the spread of genotypes that may have not been at an invaded location. *Tubastraea* spp. were recorded on at least 23 vessels related to oil production (e.g. platforms, drillships, monobuoys)^12^ of which we have analyzed samples from only five. It is highly possible that vectors not included in this study were the primary responsible for introductions along several localities in the Southwestern Atlantic. Recently, Petrobras (2016) reported that 78% of the 32 structures they operate in the Sergipe region of northeast Brazil were also contaminated with *Tubastraea* spp.

**Table 2:** Locations where the five analyzed vectors were recorded in the Southwestern Atlantic. Tc = *T. coccinea*; Tt = *T. tagusensis*.

Vector	Location (coordinates)	Year	Species	Source
P14	Caravelas field, Itajaí (26°46′2″S, 46°47′2.15″W)	2000	Tc	Identified by J. C. Creed from photographic register of Barreiros *et al*. (2000).
	Angra dos Reis, Ilha Grande Bay (23°00′53″S, 44°18′59″W)	2007	Tc	In port, J. C. Creed (pers. obs.)
	Canteiro de São Roque, Todos os Santos Bay (12°51′16″S, 38°50′17″W)	2014	Tc/Tt	In port, J. C. Creed (pers. obs.)
P27	Voador field, Campos Basin (22°22′S, 40°24′W)	2013	Tc	Identified by J. C. Creed from photographic register communicated by Ricardo Guedes dos Santos (pers. comm.).
	Canteiro de São Roque, Todos os Santos Bay (12°51′16″S, 38°50′17″W)	2014	Tc	In port, J. C. Creed (pers. obs.)
IMODCO IV	Arraial do Cabo (22°58′21″S, 42°0′49″W)	2007	Tc/Tt	Mizrahi (2008)
	São Sebastião (23°48′48″S, 45°24′11″W)	2014	Tc/Tt	In port, J. C. Creed (pers. obs.)
SBM-5 Araça	São Sebastião (23°48′48″S, 45°24′11″W)	2012	Tc/Tt	In port, J. C. Creed (pers. obs.)
FPSO Marlim Sul	Bacia de Campos (22°32′38″S, 40°01′15″W)	2016	Tc	Identified by C. Zilberberg from samples provided by the company SBM-Off-shore

Our results corroborate the hypothesis of multiple introduction events^34^, with at least five genetic clusters with no geographic pattern for *T. coccinea*. The lack of geographic pattern is likely a result of the transport of infested vectors previously discussed. However, as the software Structure is not appropriate for organisms that reproduce mainly asexually^71^, results should be interpreted with caution. Sammarco *et al*.^72^ found similar results when analyzing invasive populations of *T. micranthus* at two oil platforms in the Gulf of Mexico, with four distinct genetic clusters observed on one single platform, likely resulting from multiple introductions from distinct source populations. In addition to increasing the propagule pressure, multiple introductions can lead to an increase in genetic diversity via the isolate breaking effect by creating new genotypes, potentially benefiting invasive populations and enhancing their chance of survival^35–40^. Patterns of high genetic diversity of invaded populations associated with multiple introductions have been observed for other marine organisms, such as the green crab *Carcinus maenas*^73^, the nassariid gastropod *Cyclope neritea*^74^, the caprellid *Caprella scaura* at the Iberian Peninsula^74,75^, and others^76,77^.

NJ analyses had low support values for *T. coccinea*, showing that there is no specific structure in their distribution. Nevertheless, based on the distribution of MLG it is possible that the northern sites were invaded more than once, as they have higher genotypic diversity when compared to the southern sites. The later was invaded less than ten years ago mostly by the same genotypes, suggesting a single invasion event. For *T. tagusensis*, both Structure and NJ suggest that the Brazilian northernmost invaded site (Petroleiro do Acaraú) was colonized by a different population, not present at any other invaded site or vector analyzed herein, and is most probably the result from a single introduction event. This site is a shipwreck 1,500 km distant from Todos os Santos Bay, the closest recorded invaded site on the Brazilian coast, and the direction of sea surface currents are westward, which possibly prevents any gene flow between Petroleiro do Acaraú and the remaining Southwestern Atlantic invaded sites. Nevertheless, Mucuripe waterway terminal is ~200 km east from the shipwreck and it is possible that this invasion was also through biofouling on small or large vessels that used this terminal. The remaining invaded sites and vectors are likely derived from two different native populations. The native distribution of *T. tagusensis* is currently unknown and until an extensive revision of the genus is undertaken (Capel *et al*. in prep.) further assumptions of the origins of the Southwestern Atlantic populations are challenging.

Supporting preliminary analyses showed by Capel *et al*.^34^, we observed an excess of heterozygotes for *T. coccinea* and a higher genetic diversity on vectors when compared to invaded sites. Lineages where asexual reproduction predominates tend to have high levels of heterozygosity and negative *F*_IS_ as a consequence of an independent evolution of loci (e.g. “Meselson effect”), accumulating divergence within alleles^71,78–80^ and a higher genetic diversity on vectors in comparison to invaded sites would be expected when only a few individuals are successufully established on invaded sites. In general, the observed genetic diversity for *Tubastraea* spp. in the Southwestern Atlantic may result from a combination of factors, such as reproductive strategy, high growth rate, high propagule pressure, occurrence of multiple invasions, and through their dissemination by oil platforms and other shipping.

Understanding the invasion processes and the identification of the vectors are primordial steps for improving management and control of this ever increasing problem. Our results show that clonality and dissemination through vectors are the main reasons for the fast spread and invasive success of *Tubastraea* spp. in the Southwestern Atlantic. High clonality capability is a common feature among successful invasive species, enabling invaders with low number of individuals/low genetic diversity to reach high densities and successfully dominate the invaded region^42,56^. We also suggest that the Atlantic population was invaded more than once by different populations from the native region and that the Indo-Pacific is a possible source of the Southwestern Atlantic populations of *T. coccinea*, although a more extensive sampling of native populations and other invaded sites, such as the Caribbean, are recommended to track the exact origin of the Southwestern Atlantic invaders. We observed that vectors still hold most of the genetic and genotypic diversity and new invasions can worsen the situation by enhancing the diversity and, consequently, increase the resilience of the populations along the coast. Local strategies have been taken to control the expansion of *Tubastraea* spp. in the Southwestern Atlantic coast^81^; however, controlling the vectors responsible for introduction and dispersion is the key procedure to turn management more effective and to prevent further invasions/population expansions^82^.

## Methods

### Sampling

A total of 306 and 172 colonies of *T. coccinea* and *T. tagusensis*, from 14 and nine sites respectively, were sampled by SCUBA diving between 2012 and 2017 (Figs 1 and 2, Supplementary Fig. S3). Samples of *T. coccinea* and *T. tagusensis* were taken from eight and six invaded sites along the Southwestern Atlantic, covering the entire range of distribution of each species in the Brazilian coast, and from five and three possible vectors, respectively. The vectors include two monobuoys (IMODCO-IV, SBM-V), two oil platforms (P14 and P27) and one Floating Production Storage and Offloading Vessel (FPSO Marlim Sul). Additionally, a native population of *T. coccinea* (Taiwan) was sampled for comparison (Fig. 1). At each site, 11–27 colonies of *T. coccinea* and 6–24 of *T. tagusensis* were sampled and preserved in 96% ethanol or CHAOS buffer^83^ prior to extraction.

### DNA extraction and microsatellite amplification

Total DNA was extracted using the Phenol:Chloroform method described by Fukami *et al*.^83^. Eight and ten microsatellite markers developed by Capel *et al*.^34^ were amplified by Polymerase Chain Reactions (PCRs) for all individuals of *T. coccinea* and *T. tagusensis*, respectively. PCRs were performed in 10 μl reactions including 0.2 μM of forward primer with M13 tail at their 5′ end (TGT AAA ACG ACG GCC AGT), 0.4 μM of labeled primer (M13 with VIC, NED, PET, or 6-FAM fluorescent dyes)^84^, 0.8 μM of reverse primer, 1U GoTaq (Promega), 1X PCR Buffer (Promega), 0.20 mM dNTPs (Invitrogen), between 1.5 and 2.5 mM MgCl_2_ (following Capel *et al*. 2017), 10 μg BSA (Invitrogen), and 5–10 ng of DNA. Cycling conditions were: 95 °C for 3 min followed by 5 cycles at 95 °C, 30 s; 52–62 °C, 30 s; 72 °C, 45 s; and 30 cycles at 92 °C, 30 s; 52–62 °C, 30 s; 72 °C, 55 s; with a final extension at 72 °C for 30 min^85^. Final concentration of MgCl_2_ and annealing temperature followed Capel *et al*.^34^. Amplification was verified in 2% agarose gel. PCR products were pooled with GS600-LIZ size standard (Applied Biosystems) and genotyped in the ABI 3500 genetic Analyzer (Applied Biosystems). Genotypes were determined using the program Geneious 7.1.9^86^.

### Clonal analyses

The package ‘RClone’^87^ on R 3.2.3^88^ was used to assess the clonal structure of each species on all sites. A total of eight samples from *T. coccinea* (3%) and six samples from *T. tagusensis* (4%) failed to amplify for more than one locus and were excluded from the analyses. Of the remaining samples (298 *T. coccinea* and 166 *T. tagusensis*), 48 (16%) and 10 (6%) individuals have missing data at one locus. All individuals with identical alleles at all loci (ramets) were assigned to the same multilocus genotype (MLG, or genets). To check if individuals with the same MLG are truly clones, the probability of finding identical MLGs resulting from distinct sexual reproductive events (*P*_sex_) was calculated for each population^89^. When *P*_sex_ > 0.001, samples were considered product of distinct sexual reproduction events (not truly clones) and included in analyses of genetic diversity. Two indexes were used to describe the clonal diversity in each population, the clonal richness (*R*), taking into account the number of individuals sampled (R = (MLG − 1)/(N − 1), ranging from 0 to 1, when all samples analyzed correspond to a different MLG); and the genotypic evenness (*V*), calculated by the Simpson’s complement evenness index to evaluate equitability in the distribution of the MLG (ranging from 0 to 1, when genets each have the same number of ramets)^89,90^. For genetic diversity analyses, only unique MLGs per population were considered.

### Genetic diversity

The FSTAT program^91^ was used to test linkage disequilibrium among all pairs of loci. Subsequent analyses were done by removing loci in linkage disequilibrium with more than one other locus. The software INEst was used to evaluate the occurrence of null alleles using the individual inbreeding model (IIM) and taking into account intrapopulation inbreeding^92^. The presence of null alleles can bias several parameters usually measured in population analyses such as the inbreeding coefficient, the observed heterozigosity and fixation indexes^92^. To assess each population’s genetic diversity, the number of alleles (A), private alleles (Ap), allelic richness (Ar), observed (Ho) and expected heterozygosities (He) and were calculated using the package ‘diveRsity’^93^ in R 3.2.3^88^. The inbreeding coefficient (*F*_IS_) and deviations from Hardy-Weinberg equilibrium were calculated with the software FSTAT^91^.

### Populations structure

To explore the topology of phylogeographic relationships among sampling sites, a neighbor-joining (NJ) tree based on Cavalli-Sforza’s and Edwards chord distance, suitable for microsatellite data^94^, was constructed using the software Populations 1.2.32^95^ and the package ‘ape’^96^ in R 3.2.3^88^. To visualize possible groups of sites, a Principal Components Analysis (PCA), using sampling sites as grouping factor, was performed using the package ‘Adegenet’^97^ in R 3.2.3^88^.

To estimate the number of genetic clusters in the dataset, repeated MLGs were removed from the data set, leaving 84 and 30 individuals for *T. coccinea* and *T. tagusensis*, respectively. A Bayesian analysis was performed in the new data set using the software Structure v. 2.3.4^98^ with the admixture ancestry model, correlated allele frequency and no sampling locations as prior. The analysis was performed with an initial burn-in of 500,000 cycles followed by 500,000 additional cycles and the number of clusters (*K*) tested varied from 1 to 14 for *T. coccinea* and 1 to 9 for *T. tagusensis* with 15 iterations for each *K*-value. The most likely *K*-value was estimated by estimating the “log probability of data” for each value of *K* (mean LnP(*K*)) and Δ*K* criterion^98^ using Structure Harvester^99^.

## Supplementary information

Supplementary File

## Data Availability

The datasets generated during and analysed during the current study are available from the corresponding author on reasonable request.
