# Supplementary material for: Multiple introductions and secondary dispersion of Tubastraea spp. in the Southwestern Atlantic
Source: Sci Rep. 2019 Sep 27;9:13978. doi: 10.1038/s41598-019-50442-3 (PMC6765005; doi:10.1038/s41598-019-50442-3)
Supplement: Supplementary file 1 — Supplementary File [file 41598_2019_50442_MOESM1_ESM.docx]

**Multiple introductions and secondary dispersion of *Tubastraea* spp. in the Southwestern Atlantic**

KCC Capel^1,2,3*^, J Creed^2,4^, MV Kitahara^3,5^, AC Chen ^6^, C Zilberberg^1,2,7^

^1^ Departamento de Zoologia, Universidade Federal do Rio de Janeiro, Rio de Janeiro, Brazil

^2^ Associate Researcher, Coral-Sol Research, Technological Development and Innovation Network

^3^ Centro de Biologia Marinha, Universidade de São Paulo, São Sebastião, Brazil

^4^ Departamento de Ecologia, Universidade do Estado do Rio de Janeiro, Rio de Janeiro, Brazil

^5^ Departamento de Ciências do Mar, Universidade Federal de São Paulo, Santos, Brazil

^6^ Biodiversity Research Center, Academia Sinica, Taipei, Taiwan

^7^ Núcleo em Ecologia e Desenvolvimento Ambiental de Macaé

* [katiacapel7@gmail.com](mailto:katiacapel7@gmail.com)


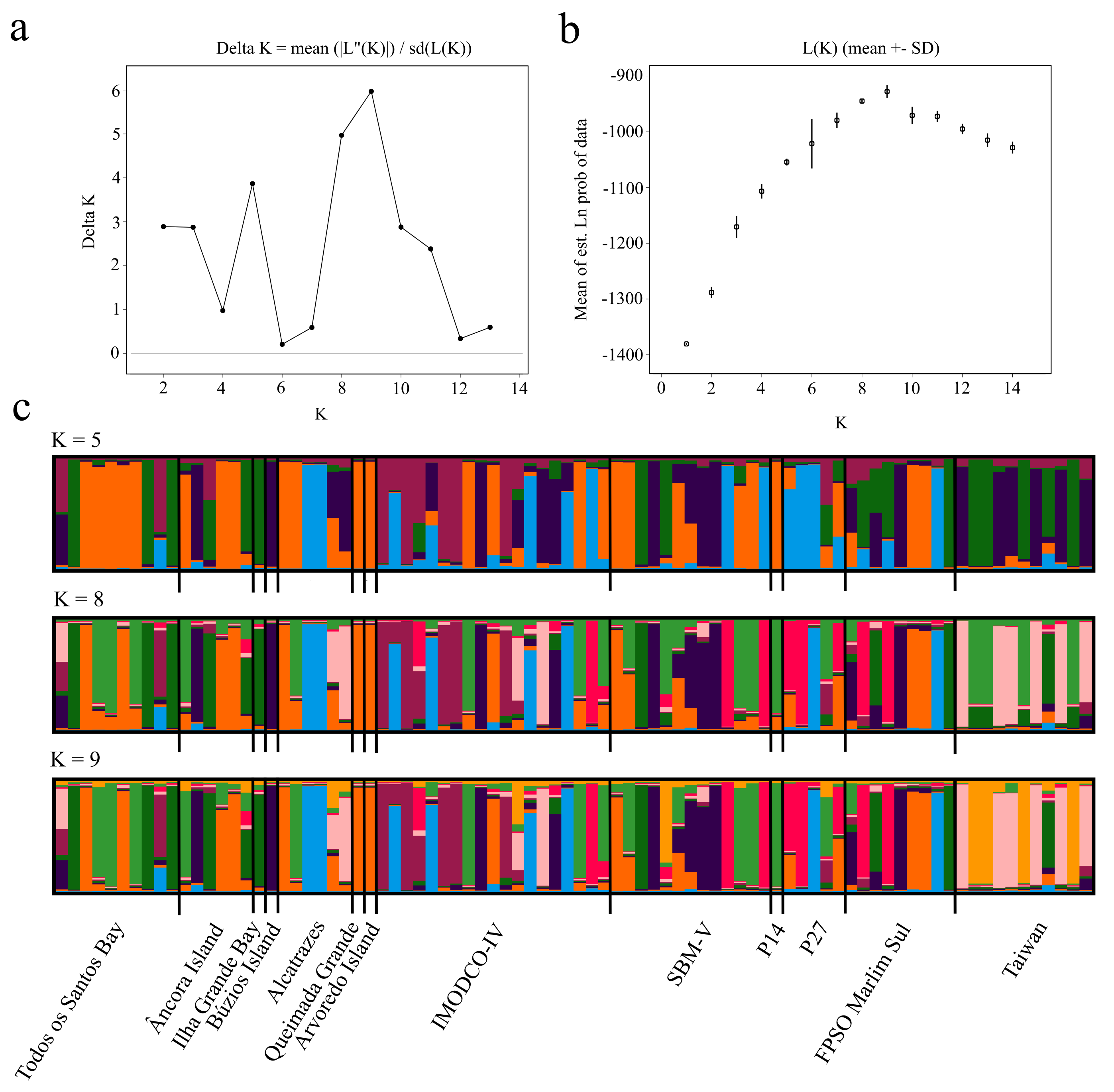


**Supplementary** **Figure S1.** Bayesian clustering analyses for *Tubastraea coccinea*. (a) shows the most likely K-value estimated by delta K and (b) estimated by the mean of estimated “log probability of data” for each value of K; and (c) shows the genetic clusters for K=5, K=8 and K=9, where each individual is represented by a vertical bar with different colors indicating the relative proportion of each genetic cluster.


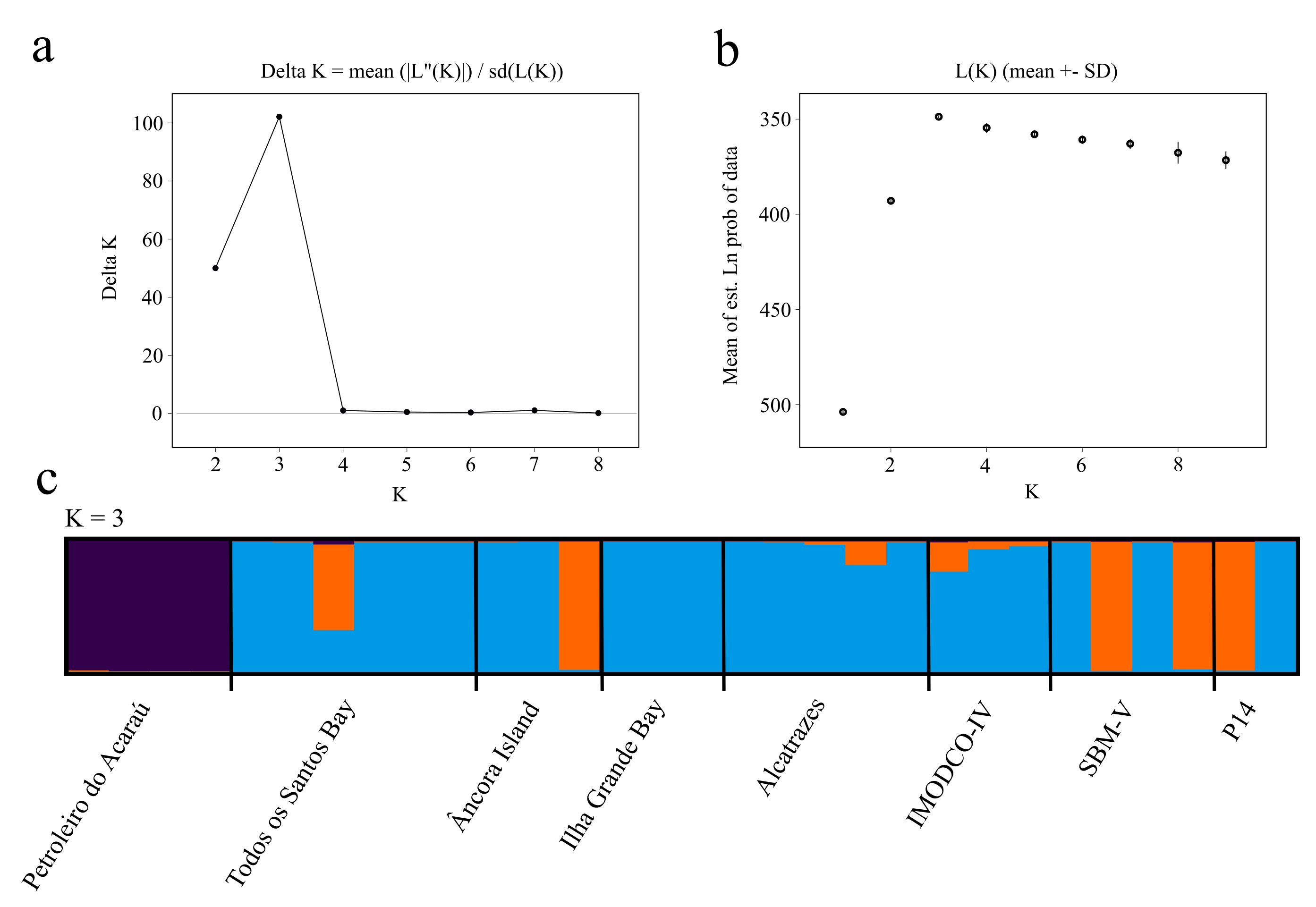


**Supplementary** **Figure S2.** Bayesian clustering analyses for *Tubastraea tagusensis*. (a) Shows the most likely K-value estimated by delta K and (b) estimated by the mean of estimated “log probability of data” for each value of K (both K=3); and (c) shows the genetic cluster for K=3, where each individual is represented by a vertical bar with different colors indicating the relative proportion of each genetic cluster.


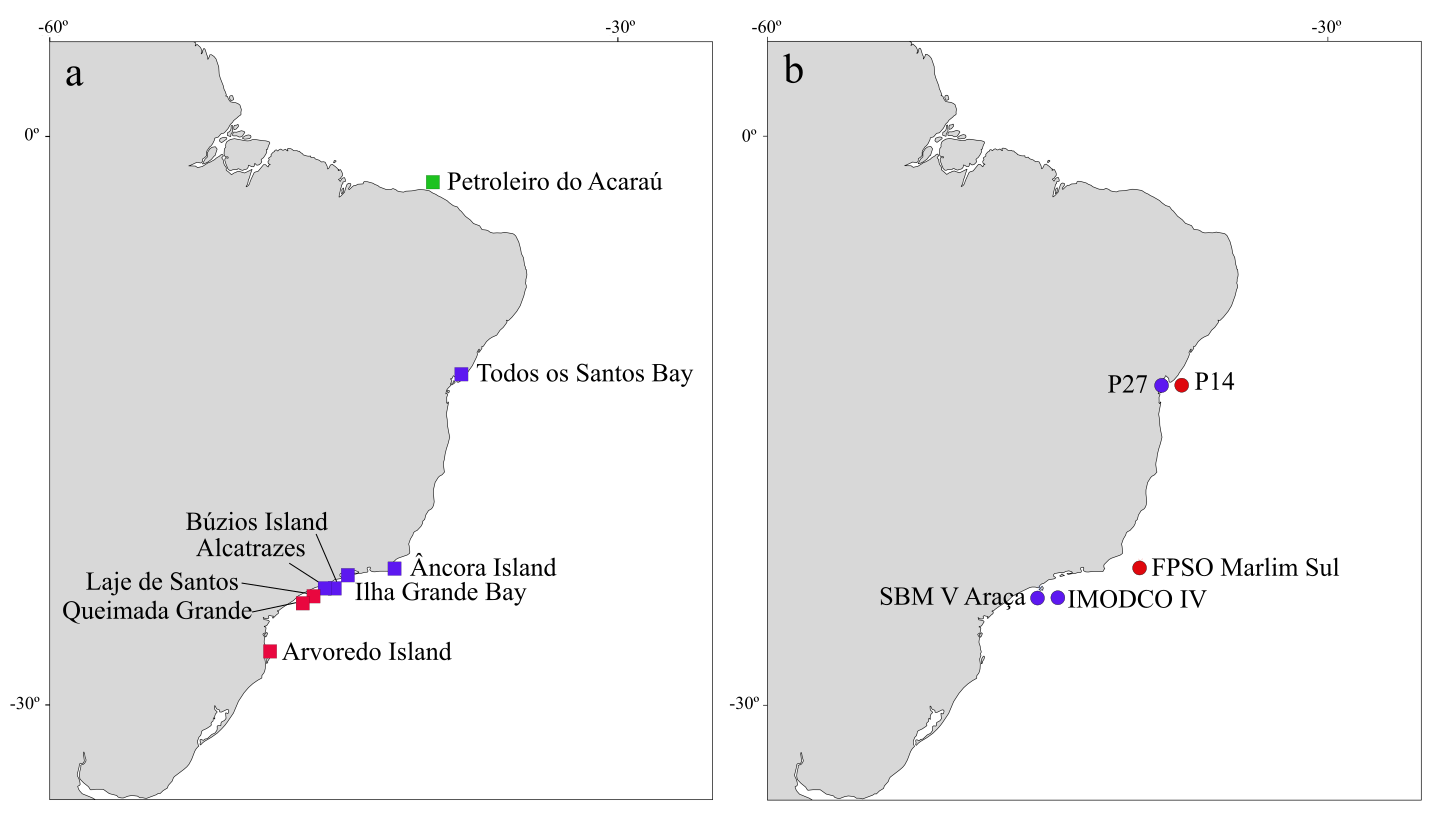


**Supplementary** **Figure S3.** Map of the sampling sites of *T. coccinea* (red), *Tubastraea* *tagusensis* (green) or both (blue) at (a) invaded sites and (b) vectors (i.e. the location of each vector when samples were taken along the Brazilian coast).

**Supplementary** **Table S1.** *Tubastraea coccinea* and *T. tagusensis* null allele frequencies for invaded, vectors and native sites. Only three loci for three different sites (showed in bold) showed frequency of null alleles ≥ 14%. One asterisk (*) indicates locus with linkage disequilibrium and were excluded from analyzes. Two asterisks (**) indicate the locus that did not amplify for *T. coccinea*.

| **Status** | **Site** | **Mean Null Allele Frequency** | | | | | | | | | |  | |  |
| --- | --- | --- | --- | --- | --- | --- | --- | --- | --- | --- | --- | --- | --- | --- |
|  |  | Tco1 | Tco4 | Tco5 | Tco8 | Tco9 | Tco29 | Tco30 | Tco34 | Tco32 | |  | |  |
| *Tubastraea coccinea* | | | | | | | | | | | |  | |  |
| Invaded | Todos os Santos Bay | * | 0.01 | 0.04 | 0.01 | 0,13 | 0.04 | * | 0.01 | | ** | | ** | |
|  | Âncora Island |  | 0.01 | 0.01 | 0.01 | 0.02 | 0.01 |  | 0.01 | |  | |  | |
|  | Ilha Grande Bay |  | 0.02 | 0.01 | 0.02 | 0.02 | 0.01 |  | 0.01 | |  | |  | |
|  | Búzios Island |  | 0.03 | 0.03 | 0.03 | - | 0.03 |  | 0.03 | |  | |  | |
|  | Alcatrazes |  | 0.01 | 0.01 | 0.01 | - | 0.01 |  | 0.01 | |  | |  | |
|  | Laje de Santos |  | 0.03 | 0.03 | 0.03 | - | 0.03 |  | 0.03 | |  | |  | |
|  | Queimada Grande |  | 0.03 | 0.03 | 0.03 | - | 0.03 |  | 0.03 | |  | |  | |
|  | Santa Catarina |  | 0.02 | 0.02 | 0.02 | - | 0.02 |  | 0.02 | |  | |  | |
| Vector | IMODCO-IV |  | 0.00 | 0.00 | 0.00 | 0.00 | 0.00 |  | 0.00 | |  | |  | |
|  | SBM-V Araça |  | 0.00 | 0.01 | 0.00 | 0.02 | 0.01 |  | 0.00 | |  | |  | |
|  | P14 |  | 0.02 | 0.02 | 0.02 | 0.02 | - |  | - | |  | |  | |
|  | P27 |  | 0.02 | 0.02 | 0.01 | 0.01 | 0.01 |  | 0.01 | |  | |  | |
|  | FPSO Marlim Sul |  | 0.02 | 0.02 | 0.01 | 0.01 | 0.01 |  | 0.01 | |  | |  | |
| Native | Taiwan |  | 0.01 | 0.01 | 0.01 | 0.07 | 0.01 |  | - | |  | |  | |
| *Tubastraea tagusensis* | | | | | | | | | | | |  | |  |
| Invaded | Petroleiro do Acaraú | 0.03 | * | 0.03 | 0.03 | - | - | **0,15** | 0.02 | | **0,14** | | * | |
|  | Todos os Santos Bay | 0.01 |  | 0.01 | 0.01 | 0.01 | 0.01 | 0.01 | 0.01 | | 0.01 | |  | |
|  | Âncora Island | - |  | 0.01 | 0.01 | 0.01 | 0.01 | 0.01 | 0.01 | | 0.01 | |  | |
|  | Ilha Grande Bay | - |  | 0.01 | 0.01 | - | - | 0.01 | 0.01 | | 0.01 | |  | |
|  | Búzios Island | - |  | 0.04 | 0.03 | - | - | 0.03 | 0.03 | | 0.03 | |  | |
|  | Alcatrazes | - |  | 0.02 | 0.02 | - | 0.01 | 0.02 | **0,21** | | 0.01 | |  | |
| Vector | IMODCO-IV | 0.01 |  | 0.01 | 0.01 | - | 0.01 | 0.01 | 0.01 | | 0.01 | |  | |
|  | SBM-V Araça | 0.02 |  | 0.02 | 0.02 | - | 0.02 | 0.02 | 0.08 | | 0.02 | |  | |
|  | P14 | 0.02 |  | 0.01 | 0.02 | - | 0.01 | 0.02 | 0.08 | | 0.01 | |  | |

**Supplementary** **Table S2.** Allelic information showing (1) the total number of alleles per locus within all sampled sites, (2) the number of alleles shared within all sampled sites and (C) the number of alleles shared between at least one site (invaded or vector) and the most divergent site, Taiwan for *T. coccinea* and Patroleiro do Acaraú (PA) for *T. tagusensis*. One asterisk (*) indicates locus with linkage disequilibrium and were excluded from analyzes. Two asterisks (**) indicate the locus that did not amplify for *T. coccinea*.

|  | Tco1 | Tco 4 | Tco 5 | Tco 8 | Tco 9 | Tco 29 | Tco 30 | Tco 34 | Tco 32 |
| --- | --- | --- | --- | --- | --- | --- | --- | --- | --- |
| *Tubastraea coccinea* |  |  |  |  |  |  |  |  |  |
| (1) Total | * | 4 | 22 | 3 | 7 | 13 | * | 7 | ** |
| (2) Shared within all |  | 2 | 0 | 2 | 1 | 1 |  | 1 |  |
| (3) Shared with Taiwan |  | 4 | 6 | 2 | 2 | 3 |  | 1 |  |
| *T*. *tagusensis* |  |  |  |  |  |  |  |  |  |
| (1) Total | 5 | * | 7 | 3 | 3 | 4 | 5 | 6 | 3 |
| (2) Shared within all | 0 |  | 0 | 1 | 0 | 0 | 1 | 0 | 1 |
| (3) Shared with PA | 1 |  | 0 | 2 | 1 | 0 | 2 | 0 | 2 |
